# Combining perspectives in multidisciplinary research on inequality in education

**DOI:** 10.1038/s41539-024-00215-z

**Published:** 2024-01-22

**Authors:** Louise Elffers, Eddie Denessen, Monique Volman

**Affiliations:** 1 University of Amsterdam, Amsterdam, Netherlands; 2 Amsterdam University of Applied Sciences, Amsterdam, Netherlands; 3 Radboud University, Nijmegen, Netherlands

**Keywords:** Education, Society

## Abstract

This comment presents some general principles for multidisciplinary research to capitalize on the growing attention for inequality in education across academic disciplines. The variety of theoretical and methodological perspectives across disciplines results in different conceptual frameworks and empirical designs to study inequality in education. While each framework and design contributes to our shared understanding of the problem, combining these perspectives requires awareness of the various lenses through which educational inequalities are being studied. We identify three dimensions along which perspectives on inequality of education vary between disciplines. These dimensions pertain to (1) how the problem of inequality in education is framed, (2) how inequality in education is empirically evaluated, and (3) how the role of education in fostering (in)equality is conceptualized. In response, we propose three general principles that may help deal with this variation when building a multidisciplinary body of knowledge on inequality in education.

Inequality in education receives growing attention in academic research across disciplines. Each discipline has its own theoretical and methodological traditions, resulting in a wide range of conceptual frameworks and empirical designs to study inequality in education. This broad scope is to be welcomed, as inequality in education is not a single-issue problem, but rather refers to a complex intertwining of mechanisms at the level of students, families, schools, neighborhoods and social, cultural, political and economic structures in societies. Hence, the building of a multidisciplinary body of knowledge, combining insights from various academic disciplines on a multitude of mechanisms underlying educational inequalities, is a promising avenue towards understanding and—eventually—tackling the problem of inequality in education.

Yet, the combination of insights from various disciplines bears the risk of resulting in a Babylonian confusion of tongues, as the conceptualization and operationalization of inequality in education vary greatly between disciplines. Especially since the problem is receiving growing attention, it is important that researchers are aware of this variety and, in response, make clear how they position their work vis-à-vis this variety of disciplinary lenses. Without such clarification, connecting research from across disciplines may obscure our broader understanding of the extent and nature of inequality in education, and the best ways to address it. In this comment, we discuss three dimensions along which perspectives on inequality of education often vary between disciplines: (1) the theoretical framing of the problem of inequality in education; (2) the empirical evaluation of inequality in education; and (3) the conceptualization of the role of education in inequality itself. In response to the variety on each dimension, we propose three principles that could help build a multidisciplinary body of knowledge on inequality of education: (1) explication of the theoretical lens, (2) consideration of the normativity of evaluations, and (3) reflection on the role of education in producing and reducing inequality.

## Theoretical framing of the problem of inequality in education

The framing of the problem of inequality is a recurring topic of academic debate, particularly in the field of philosophy. Well-known in this debate is the question what needs to be equal to speak of equality^1,2^, oftentimes resulting in a call for distinguishing equal opportunities from equal outcomes^3–6^. Does the pursuit of equality in education imply the pursuit of equal outcomes, thus problematizing unequal educational outcomes in all cases? Or does it imply the securing of equal opportunities to learn, while allowing for unequal outcomes as students may differ in their capitalizing of the opportunities provided? Another, related, debate refers to the distributive principles that should drive the quest for equality^7,8^, questioning the need for equal or unequal treatment to attain equality. Should a teacher who wishes to treat their students equally devote the same amount of time, effort and attention to each student in their classroom, or does equality require unequal treatment to cater for students’ individual needs?^9^ Various theoretical lenses can be found in research on inequality in education across disciplines. Research focusing on education as a human right (see ref. 10) tends to take the perspective of the need for equal provision of education for all^4,8^. An example is research in the field of developmental studies on equal access to quality education in developing regions^11^. In educational sciences, the provision of equal opportunities to learn—through equalizing the amount of instructional time, the curricular content or teacher quality—is a well-known approach to enhance educational equality as well^12,13^.

A different perspective stems from the fields of pedagogy and psychology, where the unequal provision of education to meet students’ individual needs and talents often serves as the starting point to examine which treatment serves which students best^8,14,15^. A well-known question in this strand of research is how educational interventions may compensate for students’ unequal starting points in life^16^. Such compensation would require an unequal distribution of educational resources, to the benefit of students from disadvantaged backgrounds. For instance, when the goal is to help all students acquire a minimum level of knowledge and skills, such as basic literacy and numeracy, unequal investment of time and attention in school would be needed to allow students from disadvantaged backgrounds to attain a required level^17^. The notion of unequal treatment to provide equal opportunities is widespread in the educational field, oftentimes with reference to the conceptual division between educational equality—suggested to require equal treatment—and equity—which is supposed to require an unequal treatment^4,14^. In contrast, research in the fields of sociology and economics often problematizes unequal treatment in education as one of the key mechanisms through which education allocates individuals to stratified positions in an unequal society^18,19^.

In response to the large variation in the framing of the problem of inequality, we identify a first principle for multidisciplinary research on inequality in education: the need to explicate the lens through which the problem of inequality in education is studied. Answering the question what needs to be equal to speak of equality helps to explicate this lens, as it makes clear whether unequal treatment and/or unequal outcomes are problematized or, in fact, strived for. As these lenses may be very different, and therefore not self-evident, awareness and explication of the theoretical framing of the problem of inequality in education enables a fruitful combining of insights and ideas from across disciplines. Review articles such as the works of Jencks^9^ and Espinoza^4^ may help researchers place their work vis-à-vis the broad range of perspectives.

## Empirical evaluation of inequality in education

Research on inequality in education typically identifies, or builds on the identification of, unequal educational outcomes between students from different backgrounds, such as achievement gaps related to gender, socioeconomic and cultural backgrounds. A group level approach problematizes unequal outcomes between student groups, and is more common in the fields of sociology^20^ and economics^21^. An individual-oriented approach, more often found in the field of psychology, focuses on the learning gains of individual students within a particular educational context^22^. From a group level perspective, equality would be obtained when group differences in student achievement are diminished. In such cases, indicators of equality are the percentages of explained variance in academic achievement scores by student background characteristics, or the performance gaps between students from different social backgrounds^23^. The individual level perspective rather focuses on equal opportunities for individual students to realize their full potential, which may result in unequal outcomes as a reflection of students’ different talents. In such cases, indicators of equality would be based on learning gains of students from different social backgrounds in particular educational settings. A frequently studied educational approach aimed at creating more educational equality from this perspective is direct instruction^24^.

Alongside differences in the extent to which unequal educational outcomes are problematized, researchers from across disciplines differ in the type of outcomes used to evaluate attempts to attain equality in education. In many cases, cognitive outcomes such as reading or math achievement (e.g. PISA, TIMSS) or diploma attainment are used to assess inequality in education. Consequently, attempts to counter inequality in education focus on cognitive outcomes. However, critical studies suggest that this focus may in fact perpetuate or even increase inequality^25,26^. A narrow focus on cognitive outcomes reinforces a hierarchy of talents and skills in which cognitive competences and abstract knowledge are valued more than practical skills and knowledge. This hierarchy frequently materializes in standardized test-based ability groups, sets, streams, or tracks. Students in the lower ranks in such hierarchical systems are aware of this hierarchy, which may negatively impact their self-esteem^27^. Standardized tests are often promoted as a means to support equality in education, as they would provide objective assessment methods and, thus, a fair selection procedure. But they are also criticized because their use may negatively affect students who do not perform well, and encourage a deficit perspective that problematizes students, families and communities^25,26^. Achievement norms based on ‘general’ standards^27^ that reflect dominant group norms^28^ may overlook knowledge and skills of non-dominant groups, thus undermining their educational opportunities. Critical perspectives on inequality, such as the ‘funds of knowledge’ approach, aim to make the curriculum more equitable and engaging for students from historically marginalized communities, by building on their knowledge, skills and cultural resources^29^. These approaches stress the need for explicit reflection on normativity in research on inequality in education.

The variation in the empirical evaluation of inequality in education brings us to a second principle for multidisciplinary research on inequality of education: consideration of the implicit norms that forms of evaluation set for the pursuit of equality in education. Such a reflection would need to include consideration of the potential reinforcement of inequality through the normativity of pursuing certain outcomes. Critical discussions such as the works of Au^25^ and Hogg^29^ may help researchers to consider the implicit norms embedded in particular evaluations and their potential repercussions for educational (in)equality.

## Conceptualization of the role of education in realizing (in)equality

Independent of the indicators used to establish inequality in education, the mere identification of inequality does not inform us about its underlying mechanisms, nor about the role education plays in decreasing or increasing inequality. Much research on inequality in education positions education as a tool to enhance equality. However, education does not only play a remedial role in leveling the playing field for learning. It can function as a catalyst for inequality just a well. Remediation and aggravation of inequality may even happen as the result of the same educational intervention. An example of this potential paradox is the topic of differentiated instruction within classrooms and differentiated curricula between tracks, which may be studied as a potential remedy for unequal achievement in one strand of research (e.g. psychology^30^), while they are studied as a catalyst for unequal achievement in a different discipline (e.g. sociology^31^). Tailoring educational programs to differences in students’ performance or needs can support students with different needs and abilities^32^. Yet, tailoring of educational programs—such as allocating students to different ability tracks and fixed ability grouping—has also been found to result in larger disparities in educational outcomes between student groups^33^. As such educational treatments guide students’ further development, it is difficult to determine to what extent a particular treatment such as tracking helped or hampered their development, especially if control groups cannot be put in place. A negative treatment effect may particularly undermine the educational opportunities of lower performing or socioeconomically disadvantaged students, as they are more often placed in specific programs with a remedial approach and less challenging standards^34^. Hence, when studying interventions to improve educational outcomes among disadvantaged or underperforming students, it is important to not just assume that these contribute to equality, but to take into account potential negative treatment effects as well.

In response to the twofold role that educational interventions may play in remedying or reinforcing inequality, we identify a third principle for multidisciplinary research on inequality of education: reflection on the potential role of education as both a producer and reducer of inequality. In some cases, educational interventions may in fact try to cure the inequality that they produce themselves^35^. Literature on the effects of educational systems or designs for various groups, such as the work of Van de Werfhorst and Mijs^18^ and Marks^27^ may inform such reflections on the potential counterfactual impact or unintended effects of interventions aimed at enhancing educational outcomes for specific groups.

## Combining perspectives in multidisciplinary research on inequality in education

In this brief comment, we reflected on three dimensions along which perspectives on inequality in education may diverge across academic disciplines. We discussed some common varieties in the theoretical framing of the problem, the empirical evaluation of inequality in education, and the conceptualization of the role of education in realizing (in)equality. In response, we proposed three general principles that may help to not only deal with, but also capitalize on these varieties in building a multidisciplinary body of knowledge on inequality in education. The first principle is: make explicit through which theoretical lens the problem of inequality in education is studied. The second principle is: consider the (norms embedded in the empirical evaluation of inequality and their consequences. The third principle is: reflect on the role that education, and some educational interventions in particular, may play in producing as well as reducing inequality in education.

Our reflection points to the importance of awareness and explication of the lens through which researchers from across disciplines study the problem of inequality in education, both in terms of their theoretical framework as well as their empirical approaches and their modeling of educational interventions. While these dimensions would be expected to interrelate, inconsistencies between theoretical and empirical approaches and the design of interventions can be found and may thus undermine our understanding of inequality and our success in tackling it^36^.

It goes without saying that this brief comment cannot do justice to all theoretical perspectives on inequality in education, nor to the rich empirical literature on the topic from all disciplines. We hope the three general principles proposed here will help researchers from across disciplines to position their work within a vastly growing field of research and establish fruitful connections between their knowledge and ideas, thus turning a vastly growing literature into a powerful multidisciplinary body of knowledge on inequality in education.

### Reporting summary

Further information on research design is available in the Nature Research Reporting Summary linked to this article.

### Supplementary information

Reporting summary
